# GAM-enhanced deepLabv3+ for accurate burn scar extraction in karst regions from remote sensing images

**DOI:** 10.1371/journal.pone.0336384

**Published:** 2025-11-11

**Authors:** Xiaodong Su, Zhijie Wang, Linzhouting Chen, Jianxing Hu, Yangsheng Wang, Shaobo Li

**Affiliations:** 1 School of Aerospace Engineering, Guizhou Institute of Technology, Guiyang, China; 2 Guizhou Unmanned Aerial Vehicle Emergency Disaster Reduction Information Engineering Research CenterGuiyang, China; 3 College of Life Science, Guizhou University, Guiyang, China; 4 State Key Laboratory of Public Big Data, Guizhou University, Guiyang, China; South China University of Technology, CHINA

## Abstract

Forest fires pose a severe threat to ecosystems, and accurate burn scar extraction is critical for post-disaster recovery and ecological management. This study proposes an attention mechanism enhanced deep learning model for semantic segmentation of burn scars in Karst regions, aiming to address challenges such as fragmented terrain and complex vegetation patterns. The model integrates ResNet50 as the backbone network to leverage its robust feature extraction capability and residual connections, mitigating gradient vanishing problem. To enhance multi-scale feature learning while avoiding grid artifacts, we optimize the Atrous Spatial Pyramid Pooling (ASPP) module by reducing dilation rates to (1, 3, 5). Furthermore, a novel Global Attention Module (GAM) is introduced after the decoder branches to dynamically recalibrate channel-spatial dependencies, enabling precise segmentation in heterogeneous backgrounds. Experiments demonstrate the model’s superiority with a mean Intersection over Union (mIoU) of 91.82% and mean accuracy (mAcc) of 95.73%, outperforming mainstream models (e.g., DeepLabV3 + , SegFormer, Mask2former) and traditional methods. The model demonstrates outstanding extraction accuracy and strong generalization capabilities; however, there remains room for optimization in terms of parameter quantity and inference speed. Future work will further explore lightweight design and real-time performance enhancement strategies. This study combines deep learning with GIS and remote sensing technology to construct a single region dataset for typical fire events in Huaxi District, Guiyang City, Guizhou Province in 2024. An efficient framework for extracting burn spots from karst landforms is proposed, which can provide real-time reference for the impact assessment, ecological restoration, and carbon flux estimation of this fire event in the region.

## 1. Introduction

Forest ecosystems are vital for maintaining the Earth’s ecological balance, regulating climate, conserving soil and water, and controlling pollution. While forest fires can promote regeneration and biodiversity under certain conditions, they often cause severe ecological and socio-economic damage [1]. Burn scars, areas that fail to regenerate post-fire, are crucial for assessing fire impacts and guiding recovery efforts [2]. Accurate identification of burn scars is essential for understanding fire distribution patterns, driving factors, and supporting fire management, prevention, and global carbon cycle research [3,4]. However, current extraction methods face significant challenges in accuracy, timeliness, and adaptability to complex terrains, particularly in Karst areas, where these limitations hinder studies of fire mechanisms and the optimization of post-disaster recovery strategies.

Karst landscapes, characterized by fragmented terrain, diverse vegetation, and ecological fragility, are highly sensitive to disturbances like fires. Studies indicate that fires in these regions can lead to long-term ecosystem imbalances, exacerbate soil erosion, and delay the vegetation recovery [5]. For instance, in Slovenia’s Dinaric Karst Mountains, fires altered forest vegetation composition and facilitated the spread of invasive species, significantly impacting ecosystem functions [6]. Additionally, post-fire phenomena such as desertification and soil acidification further complicate ecological recovery in these regions [7]. Therefore, understanding fire behavior and its ecological effects in Karst areas is crucial for improving global fire models, carbon cycle assessments and guiding fire management and ecological restoration in complex terrains.

Remote sensing technology, as a key tool for surface information acquisition, has been widely used in agriculture, forestry, environmental monitoring, and disaster assessment [8]. Advances in high-resolution, multispectral, and multi-temporal satellite imagery have made data acquisition more accessible and economical, supporting feature extraction and environmental change monitoring [9]. Since the 1970s, remote sensing has been instrumental in fire monitoring and impact assessment [10]. However, traditional burn scar extraction methods, which rely on manual interpretation or ground measurements of surface reflectance and vegetation indices, are labor-intensive, time-consuming, and less effective in complex terrains like Karst regions [11].

Recent advancements in machine learning have improved burn scar extraction through automated pattern recognition. For example, John Gajardo [12] used Sentinel-2 imagery with the Extreme Learning Machine (ELM) to classify burn scars, achieving 94% accuracy, but being limited by manual band selection. Wang [13] proposed a method using MODIS vegetation index (EVI) and land surface temperature (LST) time series data, showing high temporal precision but requiring expert knowledge and time-consuming training. Zhang [14] applied Sentinel-2 imagery with the dNBR index and Otsu threshold method to extract forest burn scars in Sichuan Province, achieving a 94% accuracy for small area but underperforming for large-scale scars. While these methods show promise, they are constrained by manual feature selection and limited generalization, particularly in complex terrains like Karst regions.

Deep learning has emerged as a powerful alternative, overcoming traditional limitations by automatically extracting features and enabling efficient remote sensing image classification [15,16]. Recent studies highlight its potential in semantic segmentation. For instance, Li [17] proposed a cross-layer detail-aware and group attention model, achieving state-of-the-art performance on high-resolution remote sensing datasets. Tang [18] improved segmentation accuracy by combining cyclic cosine annealing with multi-scale dilated convolution, outperforming mainstream models on the Gaofen Image Dataset. Jiang [19] developed a landslide disaster segmentation model using MobilenetV2 and channel attention, achieving 95.58% pixel accuracy on the Bijie City dataset. In the field of semantic segmentation, Mask2former [20], SegFormer [21], and Deeplabv3+ [22] have also achieved significant results as cutting-edge models. For example, Mask2Former uses a unique mask prediction mechanism to more accurately capture details and contextual information in images, achieving higher quality segmentation results in complex scenes. It achieved 54.4% mIoU on the COCO dataset, demonstrating strong generalization ability. SegFormer is known for its concise and efficient architecture design, which enables fast and accurate pixel level classification of images through hierarchical feature extraction and multi-scale information fusion. It achieved 55.0% mIoU on the ADE20K dataset, demonstrating its adaptability in scenes of different scales and complexities. The emergence of these models has further promoted the development of semantic segmentation technology, providing new ideas and methods for solving segmentation challenges in special areas such as ablative scar Karst regions.

Existing datasets for burn scar extraction often lack diversity and fail to capture the unique characteristics of Karst landscapes, such as fragmented terrain and diverse vegetation. Most publicly available datasets are designed for general fire-affected areas and do not account for the specific challenges posed by Karst regions, such as high spatial heterogeneity and ecological fragility. Additionally, these datasets often suffer from limited sample sizes and imprecise annotations, which hinder the training of robust deep learning models. To address these gaps, we constructed a specialized dataset using ArcGIS technology and data augmentation techniques, tailored to the unique features of Karst terrains. This dataset not only includes a diverse range of burn scar samples but also provides precise annotations, offering a robust foundation for training deep learning models to accurately extract burn scars in complex environments.

Landscape pattern-process theory [23] and restoration ecology frameworks [24] provide a theoretical basis for directly applying the results of fire spot extraction to vegetation restoration. This study proposes an efficient and accurate method for burn scar extraction in karst areas for post-forest fire assessment by combining deep learning with GIS remote sensing technology. To effectively capture the complex features of burn scars in Karst terrains, we selected ResNet-50 as the backbone network for our model, due to its optimal balance between model depth and computational efficiency. Additionally, we introduce a novel GAM (Global Attention Module) to enhance global feature interaction, addressing the limitations of traditional attention mechanisms in capturing spatial and contextual information. The main contributions include:

(1) We used ArcGIS and data enhancement techniques to construct the Fire-Scarred Landscape Semantic Segmentation Dataset (13211 images, pixel level annotations), and the samples covered typical karst vegetation types such as bare rocks, shrubs, and mixed coniferous and broad-leaved forests.
(2) We design an encoder based on ResNet50 and an enhanced ASPP (Atrous Spatial Pyramid Pooling) module can effectively capture the fire boundaries under the karst fragmented terrain and complex vegetation.
(3) We incorporate GAM into the decoder to reduce information diffusion and enhance global feature interaction, significantly improving segmentation accuracy.
(4) We have integrated GIS vectors with deep learning end-to-end to construct a fast segmentation framework for wildfire sites that balances geometric accuracy and computational efficiency.

This study presents an innovative approach for mapping fire scars in parts of Huaxi District, Guizhou Province, tackling the challenges posed by Karst terrain complexity and ecological fragility. Overcoming the limitations of existing methods, it significantly enhances monitoring efficiency and accuracy, thereby supporting fire management, ecological restoration, and global carbon-cycle research. The robust framework offers broad potential for sustainable environmental management and disaster response.

## 2. Materials and methods

### 2.1. Overview of the study area

Guiyang City (106°07′-107°17′E, 26°11′-26°55′N) is located in the central part of Guizhou Province and the Yunnan-Guizhou Plateau, and governs 6 administrative districts, 3 counties, and 1 county-level city, with a total land area of approximately 8043.37 km2. The region experiences a subtropical monsoon climate with an annual mean temperature of 15.3 °C and annual precipitation of 1197–1248 mm. Topographically, it is a typical karst mountainous landscape dominated by steep mountains and karst hills, with an average elevation of about 1180 m [25]. Slopes range from 15° to 45°, and limestone outcrops account for 30–60% of the surface, creating a fragmented terrain of peaks, gullies, and dolines that fragment the landscape and lead to highly heterogeneous micro-environments.

Vegetation is distributed as a mosaic of shrublands, Pinus massoniana stands, broad-leaved mixed forests, and Cyclobalanopsis forests, interspersed with bare rock and grass patches. This complex vegetation pattern, combined with the rugged topography, results in sharp spectral gradients and shadow effects, making burned-area boundaries indistinct and challenging to delineate.

**Fig 1:**
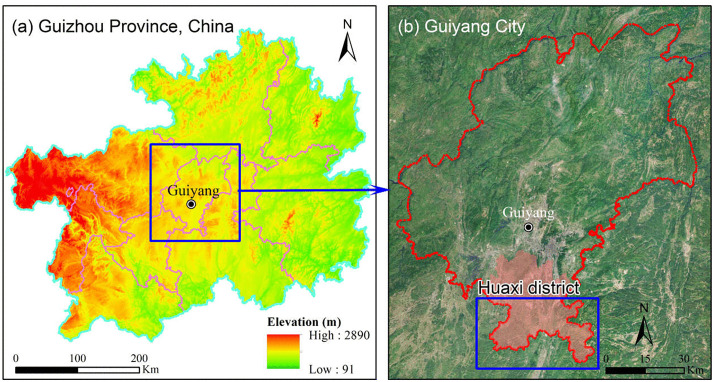
Map of the Study Area.

This study focuses on three townships in Huaxi District of Guiyang City (Yanlou Town, Maling Town, and Dangwu Town) as the experimental subjects (Fig 1). These three townships experienced a series of 9 forest fires in early 2024, which were relatively concentrated in space, covered a wide area, and caused severe damage to local forest resources, posing a serious threat to local socio-economic conditions and the security of people’s property. Additionally, drawing from the Guiyang Forest Resource Investigation Database, the affected forest types in the study area are predominantly shrub forest, Pinus massoniana forest, broad-leaved mixed forest, and Cyclobalanopsis forest. The combination of fragmented karst terrain, complex vegetation and concentrated fire events provides an ideal testbed for developing accurate burned-area extraction methods.

### 2.2. Dataset construction

To implement the research on automatic extraction of burnt areas in the Karst forest of Huaxi District, Guiyang City using deep learning, a semantic segmentation dataset of Karst forest fire scar was constructed using the Gaofen-2 (GF-2) satellite imagery captured on March 24, 2024(The remote sensing images were purchased from Anhui Digital Geographic Remote Sensing Information Service Co., Ltd).

**Fig 2:**
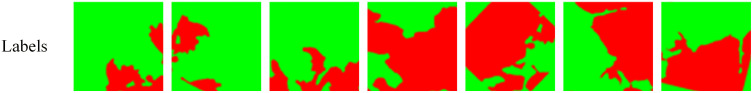
Burn Scars Labels with random rotations.

**Fig 3:**
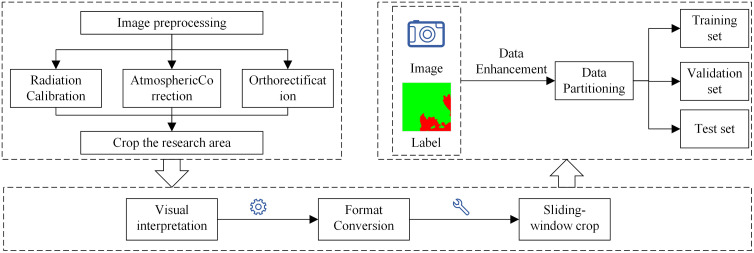
Dataset Construction Flowchart.

To accurately extract burn scar features from high-resolution remote sensing images, ArcGIS was used to visually interpret and vectorize burn scar samples images within the study area. The vector data were then converted into raster data to complete the labeling annotations. A sliding window approach was employed for cropping the images and labels to maximize the preservation of image contextual information and generate a substantial amount of training data. The cropping windows (pixels) moved with a stride of 128 pixels in each direction (50% overlap rate), forming 6610 valid sample data points. Data augmentation, aimed at increasing the diversity of the samples and enhancing model performance, was applied to the original samples through random rotations (Ranging from 0 to 360 degrees), as illustrated in Fig 2, increasing the number of samples to 13211(The probability of applying data augmentation is 100%). The samples and labels were divided into training, validation, and test sets in a 7:2:1 ratio. The method and process of dataset construction are shown in Fig 3.

### 2.3. Methods

DeepLabV3 + , proposed by Google in 2018, is a semantic segmentation model whose core idea is to fuse features of different scales to form an encoder-decoder structure. The encoder comprises a feature extraction backbone network and the ASPP (Atrous Spatial Pyramid Pooling) [26]. The backbone network uses the lightweight Xception network, whose convolution modules employ Depthwise separable convolutions to extract features and efficiently reduce the number of model parameters. ASPP, a crucial component of DeepLabV3 + , utilizes atrous convolutions with varying dilation rates to enhance the network’s receptive field and achieve multi-scale feature extraction. The decoder combines high-level and low-level features, integrating pixel location and spatial information. The fused features are then processed by a classifier for semantic recognition.

### 2.4. Burn scar extraction model based on DeepLabV3+

In this study, we developed an improved Deeplabv3 + model for precise recognition of fire-scarred images in Karst terrains. The model employs ResNet-50 [27] as the backbone network for feature extraction, which strikes an optimal balance between model depth and computational efficiency. ResNet-50 provides richer feature representations compared to shallower networks like ResNet-18, while being more computationally efficient than deeper networks such as ResNet-101 or ResNet-152. Additionally, its residual connections mitigate the vanishing-gradient problem, enabling stable training and improved performance on complex segmentation tasks. These advantages make ResNet-50 an ideal choice for handling the high spatial heterogeneity and diverse vegetation patterns characteristic of Karst landscapes. Code: https://github.com/Gitsuxd/burn-scar-extraction.

To further enhance the model’s ability to capture complex features of burn scars, we incorporated an Atrous Spatial Pyramid Pooling (ASPP) module with a smaller dilation rate of (1, 3, 5). This configuration allows the model to effectively capture multi-scale contextual information. Moreover, traditional attention mechanisms such as Squeeze-and-Excitation (SE) and Efficient Channel Attention (ECA) often struggle to effectively model global feature interactions, limiting their ability to capture spatial and contextual information. To address this limitation, we introduced a novel Global Attention Module (GAM) [28] to the model. GAM enhances feature extraction by reducing information dispersion and improving global feature interaction. It focuses on the most relevant regions of burn scars, even in highly fragmented terrains, thereby significantly improving segmentation accuracy.

**Fig 4:**
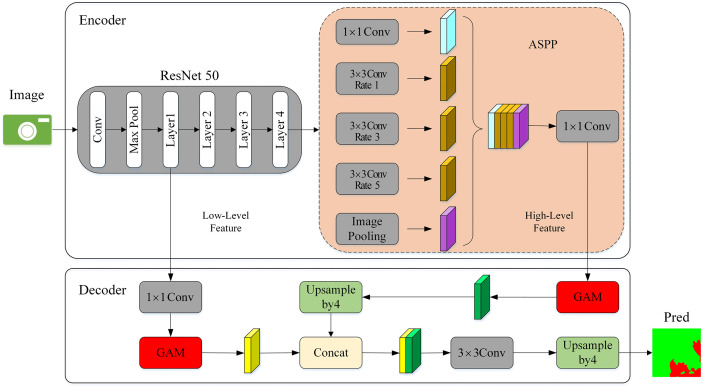
Improved DeepLabV3 + Model Structure.

The improved model structure, which integrates ResNet-50 as the backbone network, ASPP with a smaller dilation rate, and the GAM, is shown in Fig 4. This combination ensures both high accuracy and practical applicability in burn scar extraction, making it well-suited for the complex segmentation tasks associated with fire-scarred images in Karst terrains.

**Fig 5:**
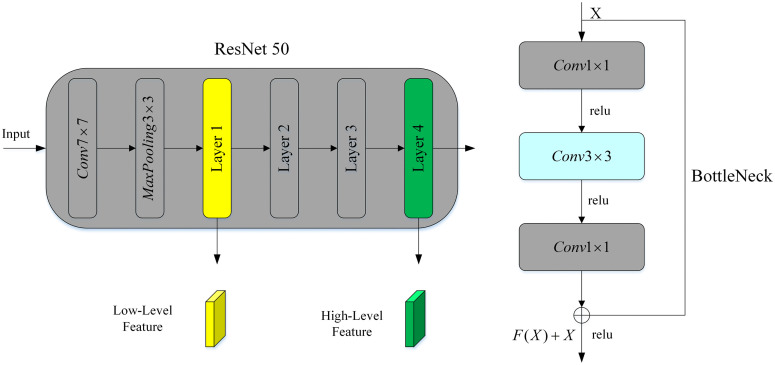
ResNet50.

In this paper, ResNet50 serves as the feature extraction network within DeepLabV3+ (Fig 5). ResNet50 is a widely-used deep residual network designed to address the vanishing-gradient problem in deep neural networks. It consists of 50 convolutional layers and employs residual connections that allow gradients to flow more efficiently during training. The architecture includes four stages of bottleneck blocks, each comprising three convolutional layers (1×1 , 3×3, and 1×1) that reduce computational complexity while maintaining rich feature representations. The output features from layers 1 and layers 4 of ResNet50 are passed through convolution layers. The feature map from Layer 1 contains more positional and detailed information, while the feature map from Layer 4 has a larger receptive field, encompassing deeper semantic and contextual information. These features are then used to support the decoder in DeepLabV3 + , with the outputs from the Atrous Spatial Pyramid Pooling (ASPP) module serving as input features for the decoder.

The ASPP is a critical component of the DeepLabV3 + semantic segmentation network. It utilizes atrous convolutions with different dilation rates to enhance the network’s receptive field and achieve multi-scale feature extraction. The ASPP module consists of four parallel convolutional layers and one pooling layer. The four parallel convolutions include one 1×1 standard convolution and three 3×3 atrous convolutions with dilation rates of 12, 24, and 36, respectively (assuming the backbone network’s down sampling factor is 8). The ASPP module fuses the results from the atrous convolutions to minimize information loss and extract contextual semantic features at various scales.

Considering the grid effects caused by high dilation rates in the ASPP module, which can lead to errors in feature extraction or missed features, the dilation rates have been adjusted to 1, 3, and 5. This adjustment addresses potential issues in the burn scar dataset, where the image size is reduced after an 8x down sampling by the feature extraction network.

**Fig 6:**
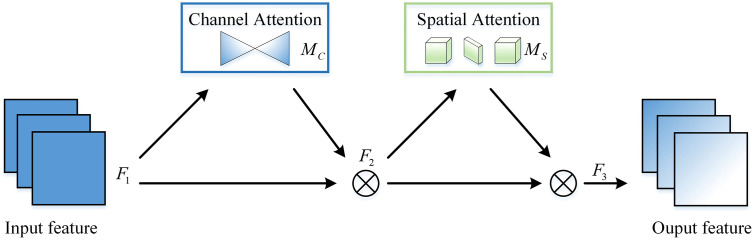
Overview of the GAM.


To enhance the model’s focus on burn scar-related features, we introduce the Global Attention Mechanism (GAM). This mechanism designed to effectively reduce information dispersion on a global scale and enhance the interaction between global features, thereby improving the model’s attention to areas of interest. The GAM attention mechanism comprises two modules: a channel attention module and a spatial attention module, as shown in Fig 6.

$$\left\{ \begin{array}{c} F_{2}=M_{c}(F_{1})⊗F_{1}\\ F_{3}=M_{s}(F_{2})⊗F_{2} \end{array}\right.$$

In the formula, $M_{c}$ and $M_{s}$ represent the channel and spatial attention maps, ⊗ represent denotes element-wise multiplication.

**Fig 7:**
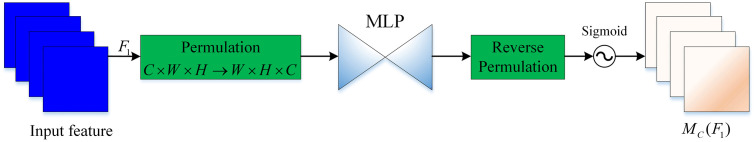
Channel Attention Module.


The GAM Attention mechanism encompasses both channel and spatial attention modules. The channel attention module uses a three-dimensional arrangement to preserve information across three dimensions. It then employs a two-layer Multi-Layer Perceptron (MLP) to amplify cross-dimensional channel-spatial dependencies. (The MLP is an encoder-decoder structure similar to BAM, with a compression ratio of r.) The channel attention submodule is shown in Fig 7.

$$\left\{ \begin{array}{c} MLP=FC(\sigma (FC(c,c/r)),c)\\ M_{C}=\delta (permute(MLP(permute(F_{1})))) \end{array}\right.$$

In the formula, c is the number of input channels, r is the compression ratio, r=4, σ is the RULE activation function, FC is the fully connected layer, *permute* is the dimension swapping function used to swap positions in different dimensions, and δ is the sigmoid activation function.

**Fig 8:**
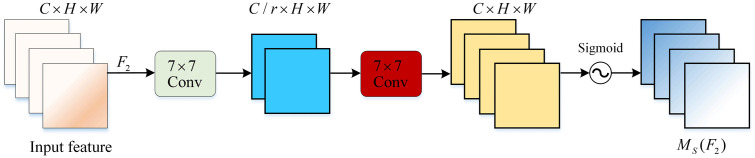
Spatial Attention Module.


In the spatial attention submodule, to focus on spatial information, two convolutional layers are used for the fusion of spatial information. The same compression ratio r used in the channel attention submodule is employed. A convolution with a kernel size of 7×7 reduces the number of channels, decreasing the computational load. A convolution with a kernel size of 7×7 increases the number of channels to maintain consistency. Finally, the output is processed through a sigmoid function. The spatial attention submodule is depicted in Fig 8.

$$M_{S}=\delta (Conv(\sigma (Conv(c,c/r)),c))$$

In the formula, c is the number of input channels, r is the compression ratio, σ is the RULE activation function, Conv is the 7×7 Convolution, and δ is the sigmoid activation function.

The Cross Entropy Loss [29] is a widely used loss function in machine learning and deep learning, particularly for classification problems. It guides the model’s training by measuring the difference between the predicted probability distribution and the true label probability distribution. In the method proposed in this paper, the normalization method for the loss function employs average loss, utilizing the average loss to guide the model training, as shown in formula (4).


$$L(Y,P)=\frac{\text{1}}{N}\sum_{i=1}^{N}L_{i}=-\frac{\text{1}}{N}\sum_{i=1}^{N}y_{ic}\sum_{m=1}^{M}\log(p_{i}^{c})$$

In the formula, N is the total number of samples; M is the number of classes (which is 2 in this paper); $y_{ic}$ is the label value (0 or 1) indicating whether the sample belongs to class c; and $p_{i}^{c}$ is the probability that the sample i belongs to class c.

### 2.5. Experimental platform

The experiments in this paper were conducted on a server platform with Ubuntu20.04 as the operating system. The hardware and software configuration were as follows: an Intel(R) Core i7-11800H @ 2.30GHz, 32GB of RAM, and an NVIDIA GeForce 3080TI GPU with 16GB of VRAM. The deep learning model is built on Python 3.9.19 and the deep learning framework PyTorch 2.3.1 with CUDA 12.2, and GPU acceleration was performed via CUDNN 8.9.6.

Training parameters: Initial learning rate: 0.01, Learning rate scheduler policy: poly, Weight decay: 0.0001, Image input size: 256×256, Batch size: 32, Iterations: 30000, Optimizer: SGD, Down sampling factor: 8, Random Seed: 1,42 and 123.

### 2.6. Evaluation metrics

This paper uses Mean Intersection over Union (mIoU) and Mean Accuracy (mAcc) as evaluation metrics.


$$mIoU=\frac{\text{1}}{n}\sum_{i=1}^{n}\frac{TP_{i}}{TP_{i}+FP_{i}+FN_{i}}$$

$$mAcc=\frac{\text{1}}{n}\sum_{i=1}^{n}{\frac{TP_{i}}{TP_{i}+FN}}_{i}$$

Where $TP_{i}$ represent the number of samples that are actually of class i and are predicted to be class i. $TN_{i}$ represent the number of samples that are not of class i and are predicted not to be class i. $FP_{i}$ represent the number of samples that are not of class i but are predicted to be class i. and $FN_{i}$ represent the number of samples that are of class i but are predicted to be another class. n represent number of classes.

## 3. Results

### 3.1. Ablation experiment

**Fig 9:**
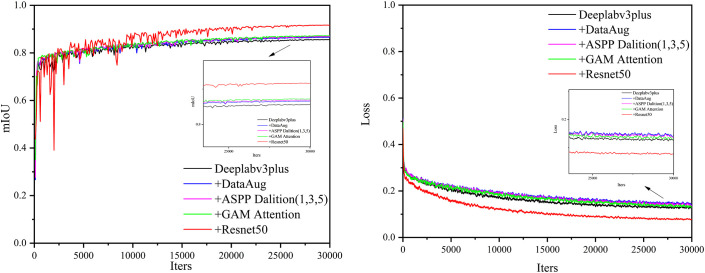
mIoU and Loss during the Training Process of the Ablation Experiment.

This study conducts ablation experiments on the fire-scar dataset with the proposed algorithm. To verify the effectiveness and soundness of each module, we report the mean ± standard deviation of mIoU and mAcc obtained with three independent random seeds. The quantitative results are summarized in Table 1, where bold numbers denote the best score for each metric. Fig 9 further illustrates the training curves of mean IoU and loss for every ablation run, with distinct colors indicating different experimental settings.

**Table 1:** Ablation Experiment on the Fire Scar Dataset.

DataAug	ASPP Dilation Rate	GAM	Backbone	mIoU mean±SD	mIoU ∆	mIoU p	mAcc mean±SD	mAcc ∆	mAcc p	Size(M)
N	12,24,36	N	Xception	85.59 ± 0.073	–	–	92.24 ± 0.005	**–**	**–**	**296.8**
Y	12,24,36	N	Xception	86.59 ± 0.045	+4.05	9.98 × 10^-29^	92.83 ± 0.025	+3.46	3.68 × 10^-21^	296.8
Y	1,3,5	N	Xception	86.91 ± 0.057	+0.39	6.35 × 10^-16^	92.99 ± 0.031	+0.25	3.49 × 10^−9^	296.8
Y	1,3,5	Y	Xception	87.25 ± 0.055	+0.77	0.0017	93.19 ± 0.040	+0.68	0.0039	310.4
Y	1,3,5	Y	Resnet50	**91.82 **± 0.113	+5.89	6.84 × 10^-108^	**95.73** ± 0.061	+4.14	1.54 × 10^-55^	332.1

a.DataAug: Data augmentation; ASPP Dilation Rate: Dilation rate of the ASPP module; GAM: GAM attention module, where N indicates that this module is not used, and Y indicates that this module is used; b.mean, SD, Δ, and p denote the average of per-image metrics across three random seeds, the standard deviation among seeds, the percentage-point increase of the latter versus the former, and the p-value from a one-tailed paired t-test, respectively.

As shown in Table 1, the base model achieves 85.59% ± 0.073% mIoU and 92.24% ± 0.005% mAcc (mean ± SD across three random seeds) on the original fire-scar dataset without data augmentation. After introducing augmentation, mIoU and mAcc increase by 1.0 and 0.59 percentage points, respectively. Paired experiments further demonstrate that mIoU and mAcc improve by 4.05 and 3.46 percentage points, respectively, with P-values of $9.98\times 10^{-29}$ and $3.68\times 10^{-21}$ ¹, strongly confirming the effectiveness of data augmentation. As illustrated in Fig 9, mIoU improves after augmentation, indicating enhanced model performance—especially in boundary delineation, which becomes more accurate. The overall rise in loss is attributed to the increased sample diversity introduced by augmentation, prompting the model to learn richer features during training and thus elevating the loss value.

Building on data augmentation, we adjusted the dilation rates in the ASPP module to 1, 3, and 5, resulting in gains of 0.32 percentage points in mIoU and 0.16 percentage points in mAcc. This modification was necessary because the training images are 256×256 and undergo 8× downsampling, reducing the ASPP feature-map size to only 32×32; excessively large dilation rates would introduce grid artifacts and lead to mis- or under-extraction. With rates set to 1, 3, and 5, we avoid grid effects while preserving ASPP’s multi-scale feature extraction capability. Paired experiments further validate this adjustment: mIoU and mAcc improved by 0.39 and 0.25 percentage points, respectively, with P-values of $6.35\times 10^{-16}$ and $3.49\times 10^{-9}$, demonstrating the statistical significance of the dilation-rate change. As shown in Fig 9, feature recognition becomes more accurate and the training loss decreases after the modification.

Before fusing high-level and low-level features, we insert a GAM attention module after the two 3×3 convolutions. This change increases mIoU by 0.34 percentage points and mAcc by 0.20 percentage points, reaching 87.25% ± 0.055% mIoU and 93.19% ± 0.04% mAcc (mean ± SD across three random seeds) while further reducing training loss. Paired experiments confirm these gains: mIoU and mAcc improved by 0.77 and 0.68 percentage points, respectively, with P-values of 0.0017 and 0.0039, demonstrating that the module significantly enhances model performance. The improvement arises because the multi-channel feature maps produced by the two convolutions contain abundant redundant information that can mislead the model; GAM strengthens focus on relevant features and suppresses the negative impact of redundancy. However, introducing GAM not only boosts performance but also increases the model size by 13.6 M, inevitably degrading real-time speed.

Building on the GAM attention mechanism, we adopt ResNet50 as the backbone for feature extraction, achieving 91.82 ± 0.113 mIoU and 95.73 ± 0.061 mAcc (mean ± SD across three random seeds). Paired experiments confirm that mIoU and mAcc increase by 5.89 and 4.14 percentage points, respectively, with P-values of $6.84\times 10^{-108}$ and $1.54\times 10^{-55}$, demonstrating that the improvement is effective and not due to chance. The performance gain is attributed to the abundant spatial hierarchical information in remote-sensing fire-scar images, which the residual structure of Resnet captures more effectively while avoiding degradation during training, resulting in a significant performance leap and a substantial reduction in loss. However, ResNet50 also increases the model size by 21.7 M, inevitably reducing real-time performance.

### 3.2. *Comparison* experiment of attention modules

A comparative experiment was conducted to verify the effectiveness and rationality of the GAM attention module on mainstream attention modules using the self-constructed fire scar dataset. The experimental results are shown in Table 2, where bold font indicates the optimal data for each metric.

**Table 2:** Comparison of Attention Module.

Method	mIoU	mAcc
ECA	86.85	93.01
SE	86.99	93.07
GAM	**87.25**	**93.19**

**Fig 10:**
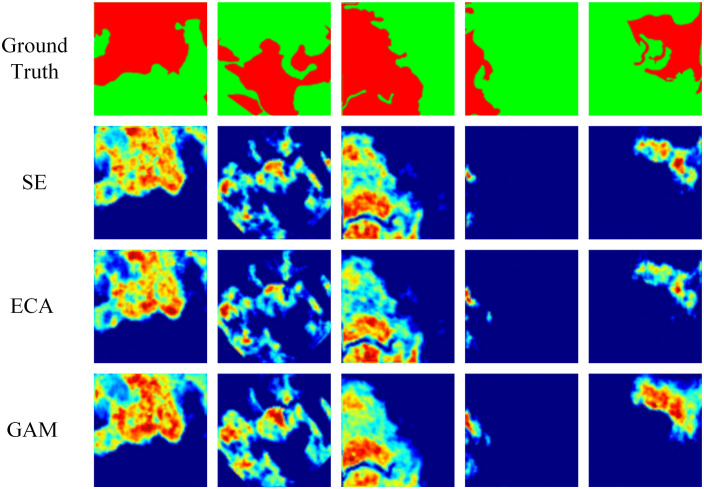
Comparison of Attention Module Visualization.

From Table 2, it can be observed that GAM achieves the best performance improvement on the fire scar dataset. Compared to ECA attention, the introduction of the GAM module results in improvements of 0.40 in mIoU and 0.18 in mAcc; compared to SE attention, the introduction of the GAM module results in improvements of 0.26 in mIoU and 0.12 in mAcc. To further validate the performance differences among different attention mechanisms, we used GradCAM [30] to visualize the heat map of the final feature map of the model. As shown in Fig 10, we can observe that GAM exhibits a more focused and precise attention distribution on fire scar areas compared to SE and ECA attention mechanisms. Specifically, the heat maps generated by GAM highlight the fire scar regions with greater intensity and consistency, while SE and ECA tend to either over-diffuse their attention to non-scar areas or under-focus on critical parts of the fire scars. This superior attention allocation by GAM directly correlates with its improved performance metrics, as it enables the model to better capture the spatial and contextual features of fire scars. Consequently, GAM effectively enhances the model’s accuracy in recognizing fire scars, as evidenced by the higher mIoU and mAcc values reported in Table 2. This visualization further supports the quantitative results, demonstrating that GAM’s attention mechanism is more adept at identifying and localizing fire scar regions compared to SE and ECA.

### 3.3. Comparison experiment with mainstream algorithms

A comparative experiment was conducted to verify the advancement of the semantic segmentation model for fire scars proposed in this paper on the fire scar dataset with Deeplabv3, Deeplabv3 + , Unet, HRNetV2, SegFormer, Mask2former et al. The experimental results are shown in Table 3.

**Table 3:** Quantitative Comparison with Mainstream Models.

Method	mIoU	mAcc	Size (M)	FPS
Mask2former-Swin-T [20]	71.20	83.23	240.3	13.9
SegFormer-B5 [21]	82.2	90.27	338.8	36.5
Deeplabv3+ [22]	86.59	92.83	296.8	6.0
Deeplabv3 [26]	90.80	95.17	317.5	5.7
Unet [31]	90.20	94.86	**99.6**	33.9
HRnetv2-W48 [32]	86.13	92.57	264.4	**50.4**
SegNext-MSCAN-B [33]	87.89	93.66	347.0	46.56
Swin Unet [34]	84.92	90.14	110.6	9.93
Ours	**91.82**	**95.73**	332.1	5.3

a. The datasets used in the above methods are all augmented datasets, and bold font in the table indicates the optimal data for each metric; b.Consistent hardware and training hyper-parameters; only iterations vary by model complexity: 30k for standard nets, 90k for heavy ones (Mask2Former/SegNext).

The quantitative comparison in Table 3 reveals significant disparities across models in terms of segmentation accuracy, complexity, and inference speed. SegFormer-B5 exhibits the lowest mIoU (80.51%) and mAcc (89.28%) despite having the highest parameter count (338.8M), which stems from its Transformer-based architecture. While self-attention mechanisms excel at modeling global dependencies, they struggle to capture fine-grained local features (e.g., fire scar boundaries) critical for remote sensing tasks, coupled with poor adaptation to spectral characteristics of satellite imagery. In contrast, Unet achieves competitive accuracy (90.20% mIoU) with minimal parameters (99.6M) through its symmetric encoder-decoder design, but its shallow structure limits multi-scale feature fusion. Deeplabv3 and Deeplabv3 + leverage ASPP modules to balance accuracy (90.80% mIoU) and moderate complexity (317.5M), yet their reliance on parallel dilated convolutions results in low FPS (5.7–6.0). HRNetv2-W48 prioritizes speed (50.4 FPS) via multi-resolution feature retention but sacrifices precision (86.13% mIoU) due to inadequate multi-scale modeling. Mask2Former-Swin further underlines the misfit of query-based instance frameworks, scoring only 71.20%/83.23% with 240.3 M and 13.9 FPS, Swin-Unet inherits hierarchical window attention but delivers 84.92%/90.14% at 110.6 M and 9.93 FPS, revealing inefficiency on high-resolution tiles, SegNext, blending MSCAN Conv-attention blocks, balances 87.89%/93.66% at 347.0 M and 46.56 FPS yet still falls short of our method.

Our method outperforms all counterparts, achieving 91.82% mIoU and 95.73% mAcc, attributable to two innovations: (1) The GAM module integrated after ASPP convolutions dynamically suppresses noise via channel-spatial attention, amplifying discriminative fire scar features. (2) The ResNet50 backbone mitigates gradient degradation through residual connections, enabling robust joint spectral-spatial learning. However, these enhancements introduce a parameter overhead of 332.1M and reduce inference speed to 5.3 FPS, primarily due to computational bottlenecks in ASPP’s multi-branch dilated convolutions and GAM’s global feature interactions. While our model achieves state-of-the-art accuracy, its practicality is constrained by high computational costs. The 5.3 FPS inference speed lags behind real-time requirements, and the 332.1M parameter size challenges deployment on edge devices.

## 4. Discussion

**Fig 11:**
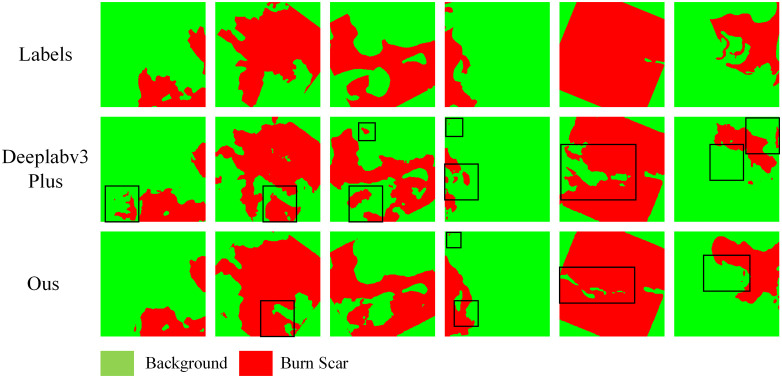
Comparison of Segmentation Results Before and After Improvement.

Fig 11 shows the segmentation results of the burn scar image before and after the model improvement. In this figure, we use the black box to mark the false detection area of the model before improvement. It is obvious that the model misclassifies some red pixels (burn scars) as green pixels (background), with a high misjudgment rate. In contrast, the misjudgment areas of the improved model are significantly reduced, and these misclassifies areas are mainly concentrated in the junction of the burned area and the bare soil or sparse vegetation area (blue box). This shows that although the improved model shows better performance, there may still be challenges in some complex backgrounds.

Furthermore, in terms of quantitative accuracy evaluation metrics, after a series of optimizations and improvements, the model’s mIoU increased from 85.59% to 91.82%, and mAcc improved from 92.24% to 95.73%, reflecting a significant enhancement in performance.

The improved model exhibits superior classification performance. The performance gains can be attributed to the following factors:

(1) Data Augmentation. By increasing the diversity of data samples, the model can learn more features, thereby enhancing its generalization ability.
(2) Smaller Dilation Rates in ASPP. This avoids grid effects and reduces issues of mis-extraction and under-extraction while still retaining the advantages of multi-scale feature extraction in ASPP.
(3) GAM Attention Mechanism. GAM enhances the model’s focus on relevant features while reducing the negative impact of redundant information on the model.
(4) Resnet Feature Extraction. Resnet allows for easier capture of the rich spatial hierarchical structural information in remote sensing fire scar images and effectively avoids degradation issues during training.

Compared with mainstream deep learning methods (Unet, Deeplabv3, HRnetV2, SegFormer, Mask2former, SegNext, Swin Unet), the model proposed in this study uses Resnet50 as the backbone network, which better captures and utilizes deep features; The introduction of attention mechanism and optimization of ASPP module enhance the model’s attention to high-level and low-level features, improve the effectiveness of feature fusion, and enable the model to have better performance on the burnt land dataset. However, the model’s real-time performance still needs improvement, and model pruning, distillation, and other work need to be carried out before model deployment.

In traditional methods of extracting burnt areas, reference [35] extracted the area of fire scars in the Kunming region based on Sentinel-2 data from 2016 to 2020, high-temperature fire point data, and land use classification data using the Differenced Normalized Burn Ratio (dNBR). Compared to visual interpretation, the average accuracy achieved was 89%. The extraction accuracy of fire scars can reach a high level over small areas, but the efficiency and accuracy of extraction over larger areas still need further improvement. Reference [36], achieving an accuracy of 96%, utilized six bands of Landsat OLI data and 14 fire-sensitive vegetation indices to implement fire scar extraction using various machine learning methods, including KNN, SVM, RF, and GNDT, was able to identify fire scars in the Muli area accurately. However, such methods require manual acquisition of core parameters, such as the number of nearest neighbors in KNN, the penalty coefficient C and RBF kernel coefficient in SVM, and the number of learners in RF and GNDT, which need to be determined through multiple preliminary experiments, resulting in poor generalization ability. The observed discrepancies among traditional methods can be attributed to three main factors. First, spectral index-based approaches (e.g., dNBR) rely on fixed thresholds that are sensitive to illumination and phenology; their performance therefore degrades under the heterogeneous illumination conditions typical of Karst terrains. Second, pixel-level machine-learning classifiers (e.g., RF, SVM) depend on hand-crafted features whose discriminative power is limited when burn-scar spectra overlap with bare soil or shadowed vegetation. Third, temporal-threshold methods (e.g., ConvLSTM with empirical change thresholds) ignore fine-scale spatial context, leading to over- or under-estimation of scar boundaries in fragmented landscapes. Our deep-learning framework circumvents these limitations by jointly learning hierarchical spectral–spatial representations and adaptive decision boundaries directly from data.

Reference [37] combined Convolutional Neural Networks (CNN) and Long Short-Term Memory networks (LSTM) to propose a spatiotemporal detection method called Stacked ConvLSTM for forest fire scar detection, achieving an accuracy of 92.4% with good detection results. However, this method relies on empirical thresholds to determine changed pixels, making its performance dependent on experience and limiting its applicability. Reference [38] proposed a fire scar extraction method based on the Unet convolutional neural network, which improved recognition accuracy by more than two times compared to traditional raster-based forest fire identification methods, with the highest accuracy estimated at 0.95 through F-measure. The above literature further illustrates that deep learning models can typically learn features automatically, reducing reliance on expert experience and the need for manual parameter adjustments. Based on deep learning the automatic fire scar identification model constructed in this paper, based on deep learning, can achieve over 96% accuracy on forest fire scar data, demonstrating higher accuracy in capturing fire scar boundaries and details. And the improved model has better performance and generalization ability than mainstream algorithms and traditional methods in the task of burning burned land.

Although our model is excellent in overall performance, there are still limitations. Specifically, in some complex scenes, such as when the fire scar is similar to the background color or the boundary is blurred, the model may be misclassified. These failure cases emphasize the necessity of introducing more contextual information or optimizing segmentation strategies in future research to improve the performance of the model in these specific situations.

Through the analysis of failure cases and misclassification of burn scars, we recognize the limitations of the model and provide the following enlightenment for future research:

(1) Further optimize the model structure: consider introducing more advanced deep learning architecture or modules to improve the model’s ability to extract and express complex features. For example, more efficient attention mechanism and multi-scale feature fusion method are explored to enhance the recognition accuracy of the model for burn scars.
(2) Enriching the training dataset: (i) implementing comprehensive data-augmentation pipelines that incorporate data from various weather conditions, lighting environments, and time periods, while applying more diverse augmentation techniques to synthetically modulate illumination spectra, color temperature and atmospheric scattering; (ii) prospectively acquiring supplementary imaging samples collected under temporally and environmentally heterogeneous conditions. Both strategies are strategically targeted at the scar phenotypes most vulnerable to inter-class confusion, thereby effectively enhancing the model’s generalization capability and temporal robustness.
(3) Combining multi-source data: In addition to remote sensing image data, other types of auxiliary data, such as meteorological data and terrain data, can be considered to provide more comprehensive information and help the model identify burn scars more accurately.
(4) Subsequent research will prioritize supplementing statistical significance analysis through methods such as cross validation, repeated training, or bootstrap to ensure the rigor and reproducibility of conclusions.
(5) Model light weighting: Including trimming redundant parameters, extracting knowledge from larger models, designing efficient modules (such as Depthwise separable convolutions), and using lightweight backbone networks (such as MobileNet) to reduce complexity while maintaining accuracy.

## 5. Conclusions

This study focuses on the Karst forest fire scars in Huaxi District, Guiyang City, and addresses the issues of low accuracy and efficiency in fire scar extraction from satellite remote sensing images. A deep learning semantic segmentation model suitable for Karst regions is proposed for the rapid and precise identification of fire scars in remote sensing images.

(1) A remote sensing image dataset for forest fire scar semantic segmentation was constructed using ArcGIS and data augmentation techniques, expanding the original data to increase the diversity of the dataset and providing reliable data support for improving model generalization ability.
(2) The proposed model employs the classic ResNet50 network as the feature extraction backbone, utilizes lower dilation rates in ASPP, and introduces the GAM attention module to enhance the model’s feature fusion capability and focus on fire scar features.
(3) The improved model demonstrates outstanding performance in identifying fire scar imagery, achieving a mean Intersection over Union (mIoU) of 91.82% and a mean Accuracy (mAcc) of 95.73%. This model provides an efficient computer vision solution for fire scar monitoring and analysis in the karst region of Guiyang, Guizhou Province. It offers valuable insights for post-disaster management and forest fire prevention engineering, while also supplying critical data support for global carbon cycle research. The generated high-resolution fire scar maps can be directly imported into GIS to prioritize restoration areas, optimize resource allocation, and monitor vegetation recovery. This enables ecological synergy between automated extraction and on-the-ground restoration efforts, holding significant scientific and practical value.
